# Predictors of Urinary Pyrethroid and Organophosphate Compound Concentrations among Healthy Pregnant Women in New York

**DOI:** 10.3390/ijerph17176164

**Published:** 2020-08-25

**Authors:** Arin A. Balalian, Xinhua Liu, Eva Laura Siegel, Julie Beth Herbstman, Virginia Rauh, Ronald Wapner, Pam Factor-Litvak, Robin Whyatt

**Affiliations:** 1 Department of Epidemiology, Mailman School of Public Health, Columbia University, New York, NY 10032, USA; aa3794@columbia.edu (A.A.B.); els2205@cumc.columbia.edu (E.L.S.); 2 Department of Biostatistics, Mailman School of Public Health, Columbia University, New York, NY 10032, USA; xl26@cumc.columbia.edu; 3 Columbia Center for Children’s Environmental Health, Department of Environmental Health Sciences, Mailman School of Public Health, Columbia University, New York, NY 10032, USA; jh2678@cumc.columbia.edu (J.B.H.); var1@cumc.columbia.edu (V.R.); rmw5@cumc.columbia.edu (R.W.); 4 Heilbrunn Department of Population and Family Health, Mailman School of Public Health, Columbia University, New York, NY 10032, USA; 5 Department of Obstetrics and Gynecology, Columbia University, New York, NY 10032, USA; rw2192@cumc.columbia.edu

**Keywords:** organophosphate, pyrethroid, pesticides, predictors, modeling strategies, limit of detection

## Abstract

Our study aimed to investigate dietary and non-dietary predictors of exposure to pyrethroids, organophosphates pesticides and 2,4-D herbicide in two cohorts of pregnant women in New York City: 153 women from the Thyroid Disruption and Infant Development (TDID) cohort and 121 from the Sibling/Hermanos Cohort(S/H). Baseline data on predictors were collected from the women at time of recruitment. We used three different modeling strategies to address missing data due to biomarker values below the limit of detection (<LOD): (1) logistic regression models with biomarkers categorized as (<median, ≥median); (2) linear regression models, imputing the <LOD values with (LOD/√2); (3) regression models, considering <LOD values as left-censored. Generally, all three models identified similar predictors of exposure. We found that ethnicity, higher income and education predicted higher concentrations of most of the biomarkers in both cohorts. Mothers who consumed processed meat in the TDID cohort, and broiled, barbequed food or burgers in the S/H cohort, tended to have lower concentrations of organophosphates and 2,4-D. The choice of modeling led to a few different predictors identified, and the selection of modeling strategy should be based on the study question.

## 1. Introduction

Organophosphates and pyrethroids are insecticides that are widely used for agricultural purposes while pyrethroids are also used for residential pest control and personal protection [1,2,3]. While many were registered from 1960–70, their use increased following prohibition on use of persistent organochlorine pesticides such as dichlorodiphenyltrichloroethane (DDT) in most of the world. Unlike the organochlorines, both organophosphates and pyrethroids are considered non-persistent insecticides. Although pyrethroids tend to bioaccumulate in aquatic environment, they generally have short biologic half-lives and degrade rapidly in most of the outdoor environments, but not indoor environments [4,5,6]. As such, they do not bioaccumulate and they must be applied frequently in order to be effective [2].

A main source of exposure to these compounds is diet [7]. However, residential application of the pesticides [8,9] is also an important source of exposure, as many US households use pesticides for pest control [10,11]. Organophosphates were widely used for this purpose until they were banned for residential use in 2001–02 [12,13]. Pyrethroid use was unimpacted by this regulation. On the other hand, the herbicide 2,4-D has been one of the most commonly used conventional herbicides in the home and garden market sector from 2008–2012 [14]. Although 2,4-D was detected in 78.9–84.5% of drinking water as reported by United States Department of Agriculture (USDA) between years 2008–2010 [15,16,17], it was not detected in the drinking water in New York City during the same period [18,19,20]. Nonetheless, 2,4-D is one of the main herbicides used in New York city’s parks and highways for vegetation control [21].

Organophosphates and pyrethroids have different mechanisms of action. Organophosphates bind with enzymes such as acetylcholinesterase to inhibit neurotransmitter-based synaptic transmission [22], while pyrethroids impede the opening and closing of sodium channels in insect and mammalian nerve and muscle cells [23,24]. In humans, these chemicals are metabolized rapidly by the body. As the organophosphates and pyrethroids or their metabolites are promptly excreted in the urine, it is the optimal matrix to measure exposure [25,26]. For example, diazinon and chlorpyrifos, two organophosphates, are metabolized to 2-isopropyl-6-methyl-4-pyrimidinol (IMPy) and 3,5,6-trichloro-2-pyridinol (TCPy) [27]. The half-lives for diazinon and chlorpyrifos are 2.5–5 h in rats and up to 3 days in humans, respectively [28,29]. 3-phenoxy benzoic acid (3-PBA) is a metabolite of a number of pyrethroids, including permethrin, deltamethrin, allethrin, cypermethrin, resmethrin and fenvalerate [30,31,32]; they may persist in fatty tissues with a half-life of 4–5 days [24]. In contrast, a number of non-persistent herbicides, such as 2,4-dichlorophenoxyacetic acid (2,4-D), are not metabolized and are excreted unchanged [33]. The half-life of 2,4-D is between 10–20 h in living organisms [34].

Although these non-persistent pesticides (NPPs) have short half-lives, long-term repeated exposure leads to chronic exposure which may be associated with impaired fetal growth [35,36,37] as well as neurobehavioral and developmental adverse effects [37,38,39,40,41]. Given the widespread adoption of pyrethroids and organophosphates, the potential risk of adverse health effects is cause for concern, and has already resulted in the ban on chlorpyrifos and diazinon for residential use in the US in 2001 and 2002, respectively [12,13].

As these NPPs may disrupt fetal growth and subsequent child development, it is prudent to evaluate the predictors of exposure to aid in the identification of those at greatest risk. Although various studies have been conducted to examine selected dietary and non-dietary predictors of exposure, findings are inconsistent. With respect to non-dietary predictors of exposure, some studies found that proxies for higher socioeconomic background such as higher education [37,42], home ownership [23], and being married [42] were associated with higher concentrations of metabolites of pyrethroid pesticides. Other sociodemographic predictors of exposure identified by researchers include age, race and tobacco use [23,42,43]. Higher maternal BMI [37] and adult BMI [44] were also found to be associated with higher concentrations of the pyrethroid permethrin both in children and adults, respectively. Self-reported pesticide use was found to be associated with higher concentrations of permethrin biomarkers in a study in Washington [43]. Nonetheless, reported household pesticide use was not associated with urinary 3-PBA at any age group in a study of National Health and Nutrition Examination Survey (NHANES) 1999–2002 subsample [23].

With respect to dietary predictors of exposure, consumption of vegetables and fruits was associated with higher concentrations of some of these organophosphates [45,46] and pyrethroids [47], while self-reported consumption of organic food was associated with lower concentrations of organophosphate metabolites [48,49,50,51].

The different studies do find different predictors of these pesticide exposures and there may be a variety of explanations for these seemingly discrepant findings. First, we suspect that concentrations of organophosphates may be declining from 2000 due to the aforementioned residential bans on several compounds [14]. Thus, study year may drive the proportion of participants with either low concentrations or those under the limit of detection.

Second, the laboratory assays used may differ in the limits of detection (LOD). For example, low concentrations of these NPPs may be undetectable by currently available technology (or by what was available at the time of the study), complicating data analysis [44,52,53,54] by leading to problems in transportability of findings. Individual studies have taken different approaches to address the issue of observations with values lower than the LOD. While some studies have replaced the values <LOD with $\frac{LOD}{2}$ [44,45,46,55,56] or $\frac{LOD}{\surd 2}$ [37,45,57], others have treated these observations as left-censored [23] or have not included them in the analysis [42,47].

Third, studies are limited by the data that they measure; for example, [45] some may not have fully measured nutritional intake. Fourth, results may rely on the statistical methods used; for example, most of the studies examining exposure predictors have used linear regression [37,45,46,56] while several others have used Tobit linear regression [23,54] and logistic regression [42,54,55] to determine the predictors of NPPs. Finally, we acknowledge that populations may indeed have different predictors due to their unique situations.

In this study, we use three different modeling strategies in an attempt to capture the relationship between dietary and non-dietary predictors of exposure to pyrethroids and organophosphates and 2,4-D herbicide in two cohorts of healthy pregnant women living in New York City. The modeling strategies aim to account for undetectable levels of these NPPs and to clarify differences in results obtained given different modeling decisions. We also demonstrate how study hypotheses inform modeling decisions and strategies used to address left-censored observations.

## 2. Materials and Methods

### 2.1. Study Design

We used two US pregnancy cohorts from the same geographic area and delivery hospitals to examine and compare the predictors of organophosphates and pyrethroids: the Thyroid Disruption and Infant Development (TDID) Study [58] and the Sibling/Hermanos (S/H) Birth Cohort [59]. Details regarding these cohorts, as well as their follow-up, are described elsewhere [58,59]. TDID enrolled pregnant women as they presented for prenatal care and followed their offspring through birth and early childhood. TDID recruited 316 women in their first or second trimester to participate in the study between September 2009 and December 2010. At the time of their first study visit, blood and urine samples were collected from the mothers. Pesticide levels were measured in maternal urine if measures of neurodevelopment were available for the child at age three and if there was a sufficient amount of urine for analysis (*n* = 153). The mothers for whom the pesticides’ levels were measured did not differ on sociodemographic characteristics from the rest of the cohort in terms of demographic characteristics (data not shown, available upon request).

The participants in the S/H cohort were pregnant women who had previously been enrolled in the Mothers and Newborns Study [60] and who were invited at the beginning of 2008 to enroll in the Sibling/Hermanos Birth Cohort during their subsequent singleton pregnancy (*n* = 121) [59]. Maternal urine samples from all the S/H cohort participants were collected during the third trimester, and the concentrations of the pesticides were measured.

The S/H cohort participants were residents of the upper Manhattan and South Bronx areas in New York City [59]. TDID enrolled healthy pregnant women from various prenatal clinics in different areas of New York City [58]. Both cohorts have a similar proportion of women of Hispanic origin, but the cohorts differed in terms of racial composition (Table 1).

### 2.2. Ethical Statement

The Institutional Review Board (IRB) of Columbia University approved this study protocol. All the participants provided written informed consent. The consent included a statement that all data presented for publication would be grouped, rather than individual.

### 2.3. Urinary Concentrations of Organophosphate and Pyrethroid Pesticide Metabolites

Concentrations of organophosphates and pyrethroids were measured in the Pesticides Laboratory at the Division of Laboratory Sciences at the Centers for Disease Control and Prevention. All analyses were performed as reported previously [61]. In total, 274 urinary samples from pregnant women in the TDID and S/H cohorts were analyzed. We assessed urinary concentrations of metabolites of three organophosphates and pyrethroids, 2-isopropyl-6-methyl-4-pyrimidinol (IMPy) and 3,5,6-trichloro-2-pyridinol (TCPy), para-Nitrophenol (PNP), 3-phenoxybenzoic acid (3-PBA), 4-fluoro-3-phenoxybenzoic acid(4-F-3-PBA), trans-3-(2,2-Dichlorovinyl)-2,2-dimethylcyclopropane carboxylic acid (trans-DCCA)and metabolites of two herbicides 2,4-dichlorophenoxyacetic acid (2,4-D) and 2,4,5-Trichlorophenoxyacetic acid (2,4,5-T). The limits of detection (LOD) were 0.15 (µg/L) for 2,4-D and 0.1(µg/L) for the TCPy, IMPy, 3-PBA. Nonetheless, due to large number of lower than LOD values (84–100%), 2,4,5-T, 4FP and trans-DCCA were excluded from this analysis. We also did not include PNP as it is a not a specific metabolite of methyl and ethyl parathion along with other non-pesticide compounds such as nitrobenzene and both methyl and ethyl parathion are strictly restricted in the United States [62]. We also measured the concentrations of creatinine (mg/dL) in urine by methods described previously [63].

### 2.4. Assessment of Predictor Variables

A detailed description of the exposure assessment instrument for TDID is described elsewhere [58]. Briefly, we administered a structured questionnaire to assess the demographic, lifestyle, and dietary characteristics of the study participants. The dietary questions focused on dietary habits from 6 months before conception and throughout pregnancy. The total dietary consumption was recorded based on the frequency of consumption of the food items throughout pregnancy. Dietary questions assessed the consumption of dairy, fish, beef, pork, chicken, and fast food, as well as organic vegetables. Lifestyle characteristics included employment, home ownership status, maternal education, household income, marital status, race, ethnicity, pre-pregnancy weight, and height.

The pregnant women in the S/H cohort answered a 45 min-long structured questionnaire during the third trimester, similar to the Mothers and Newborns Study [60]. The questionnaire included general demographic, gardening and household pesticide use, and questions related to general dietary habits during pregnancy. The questionnaire used in S/H did not include questions regarding vegetable and fruit consumption; however, it included questions regarding types of meat (e.g., poultry, beef, pork, or sausage) and cooking methods (broiled, fried, smoked, boiled, or barbecued). The survey also included questions regarding the consumption of smoked foods (e.g., nuts, or fish). We further attempted to harmonize common sociodemographic variables by combining similar categories (Table S1). We used the predictor variables with harmonized categories for our analysis.

### 2.5. Statistical Analysis

In total, 153 participants from TDID and 121 participants from S/H were included in the study. We performed all the analysis using SAS statistical software (SAS Institute INC., Cary, NC, USA) [64]. In order to account for urinary dilution, we divided the pesticide concentrations by urine creatinine concentration(mg/dL) [65]. The mean, median of the detected observations, and percent of the observations <LOD were calculated for TCPy, IMPy, 3-PBA, 2,4-D. Sample characteristics were described using frequencies/proportions for categorical variables and mean and SD for continuous variables. We compared the sociodemographic characteristics of the TDID and S/H participants using Wilcoxon rank-sum test for continuous and chi-square tests for categorical variables.

As the urinary biomarkers had values below the limit of detection (<LOD) for 3.92 to 59.47% of observations, we used three different strategies to address this issue and assess the associations between dietary and sociodemographic factors and maternal urinary concentrations of the five chemicals.

The first strategy dichotomized the urinary markers of pesticides at the median after adjusting for urinary creatinine in each cohort to assess the associations between odds of exposure above median and dietary and non-dietary predictors. In this strategy, values <LOD were included in the below the median category. The backward elimination method for logistic regression was used to identify a set of predictors under the condition that they were associated with the outcome at *p* < 0.1. Associations between the set of predictors and each metabolite were estimated using proc logistic procedure in SAS 9.4 (MODEL A).

The second strategy replaced the values <LOD with $\frac{LOD}{\surd 2}$ as described previously [66]. The method assumes that the biomarker follows a log-normal distribution and shape of the distribution for values between zero and LOD can be approximated by a right triangle [66]. We fit linear regression models to examine the predictors of the log-transformed concentrations of TCPy, IMPy, 3-PBA, 2,4-D adjusted for urinary creatinine. The backward elimination method for variable selection with linear regression was used to identify a set of predictors that were associated with the outcome at *p* < 0.1. (MODEL B).

The third method also considered that the chemicals below LOD have true values between zero and LOD. We used the nonparametric maximum likelihood method to estimate the cumulative distribution function for each urinary creatinine adjusted pesticide variable with the data subject to left censoring (Turnbull, 1976) and selected a parametric model to approximate the empirical distribution such that the parametric estimates were within the 90% confidence band of the non-parametrically estimated distribution curve. In TDID cohort, Weibull distribution was used for TCPy, and the log-logistic distribution was used for the remaining chemicals. In the S/H cohort, the Weibull distribution fit TCPy best, the Gamma distribution was used for 2,4-D, and the log-logistic for the other three chemicals. Using the technique of backwards elimination method for variable selection, final regression models for each chemical kept the dietary and sociodemographic factors that were associated with the outcome at *p* < 0.1 (MODEL C). The analysis used proc lifereg procedure in SAS 9.4.

## 3. Results

**Figure 1:**
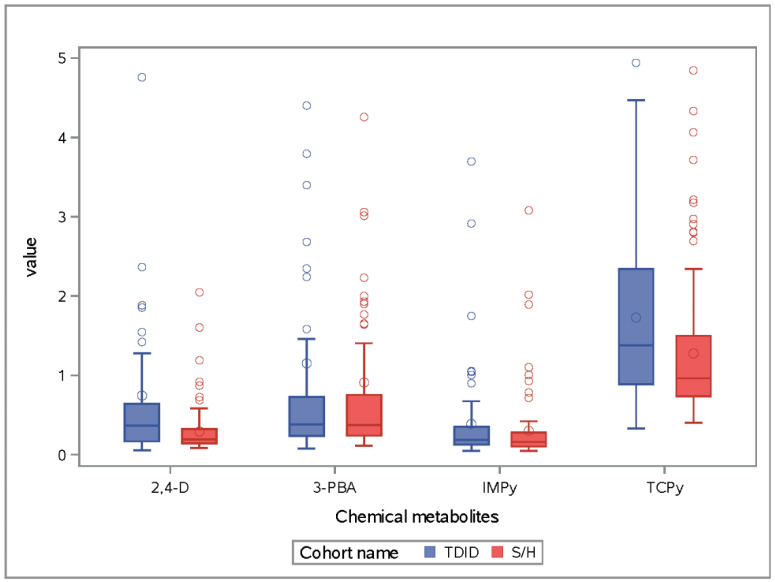
Comparison of concentrations of four metabolites of organophosphate, pyrethroid pesticides and 2,4-D herbicide by cohort.

**Figure 2:**
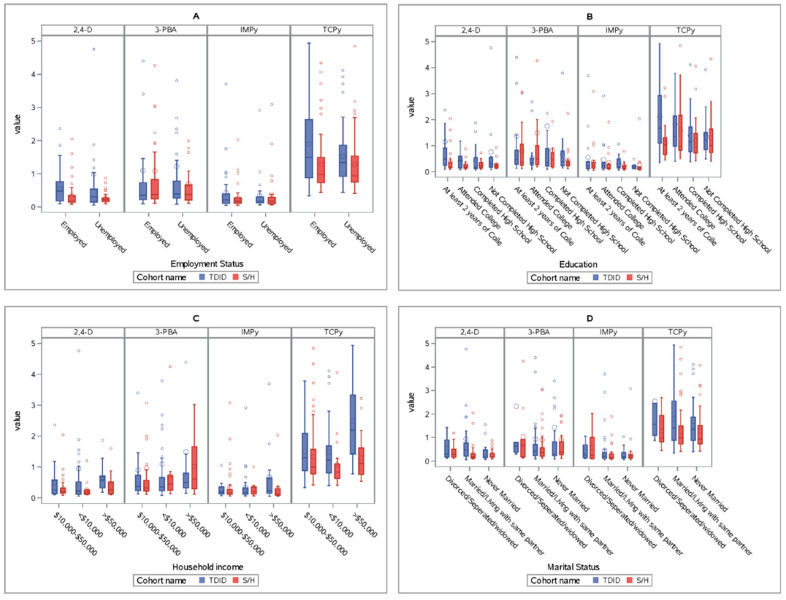
Comparison of four metabolites, 2,4-D, 3-PBA, IMPy, and TCPy, by cohort and by baseline sociodemographic characteristics of TDID and S/H cohort participants. (**A**) Comparing concentrations of 2,4-D, 3-PBA, IMPy and TCPy in the S/H and TDID cohorts in terms of employment status. (**B**) Comparing concentrations of 2,4-D, 3-PBA, IMPy and TCPy in the S/H and TDID cohorts in terms of maternal education. (**C**) Comparing concentrations of 2,4-D, 3-PBA, IMPy and TCPy in the S/H and TDID cohorts in terms of household income. (**D**) Comparing concentrations of 2,4-D, 3-PBA, IMPy and TCPy in the S/H and TDID cohorts in terms of marital status.

Demographic characteristics comparing the TDID and S/H cohorts are shown in Table 1. The participants in the two cohorts did not differ in terms of maternal age, maternal pre-pregnancy BMI, maternal education and employment status, and home ownership (Table 1). The TDID participants with available measures of TCPy, IMPy, 3-PBA, and 2,4-D did not differ from those with missing measures in terms of race, ethnicity, marital status, home ownership status, household income, employment status, education, maternal age, and maternal pre-pregnancy BMI (Table S2). The proportion of participants having <$10,000 household income in TDID cohort was significantly higher than the S/H cohort. The distribution of TCPy, IMP, 3-PBA, and 2,4-D in the two cohorts are shown in Table 2 and illustrated in Figure 1. Except for TCP-y the median and geometric mean were similar in the S/H cohort and the TDID cohort for the rest of the metabolites (Figure 1, Figure 2 and Figure S1). Both TDID and S/H cohorts also had higher geometric means and medians compared to national data in NHANES 2001–2002 and 2009–2010 (Table S3) [67].

**Figure 3:**
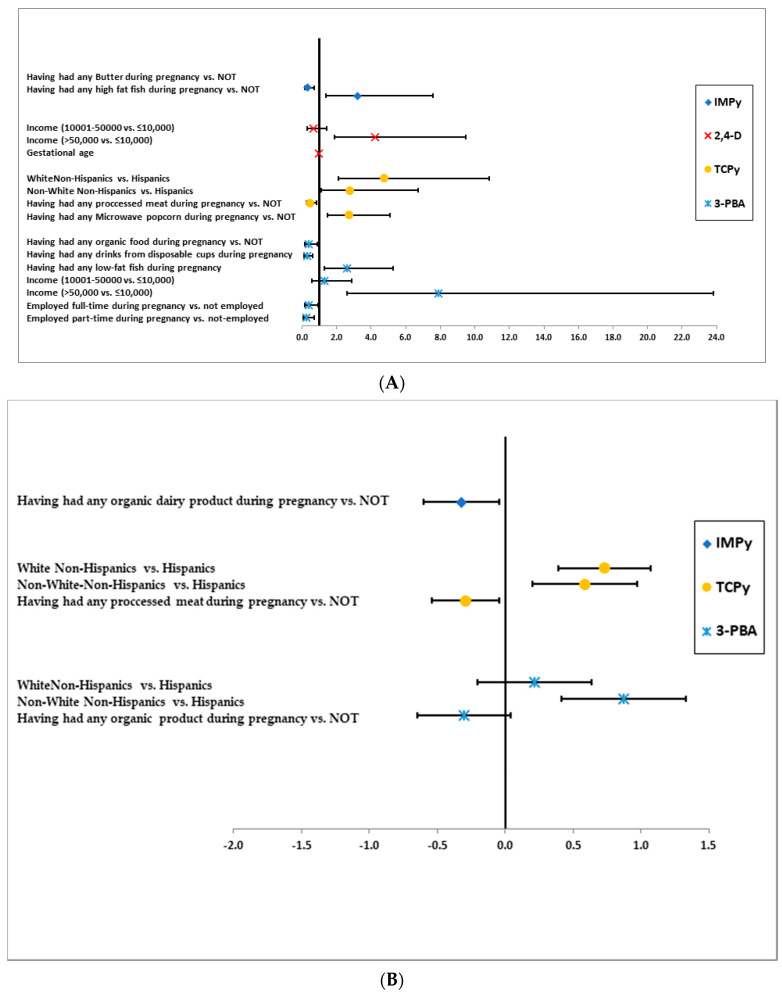

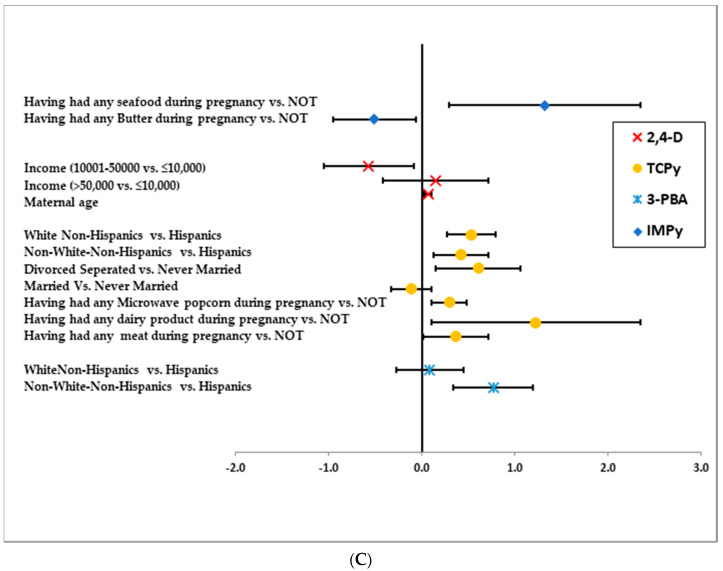
(**A**)**.** Predictors of Concentrations of organophosphate and pyrethroid pesticides in TDID cohort identified with logistic models (≥median vs. <median [Reference category]) MODEL A. (**B**)**.** Predictors of Concentrations of organophosphate and pyrethroid pesticides in TDID cohort identified by linear model with < LOD imputed by/√2 (MODEL B). (**C**)**.** Predictors of concentrations of organophosphate and pyrethroid pesticides in TDID cohort identified by regression models with lower values censored by LOD. * No predictors were selected by backward elimination procedure with the model for 2,4-D.

**Figure 4:**
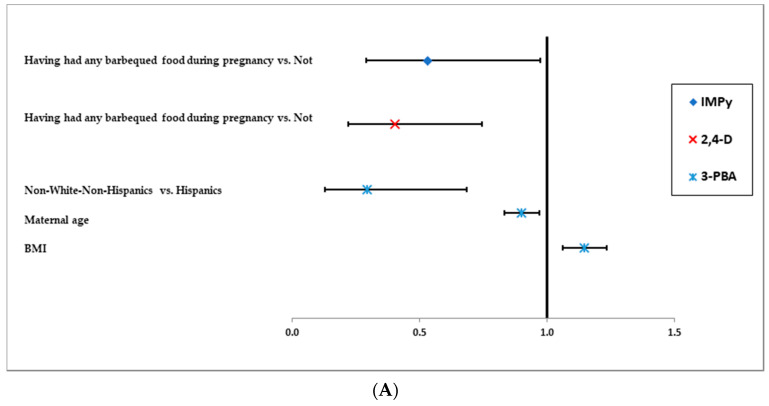

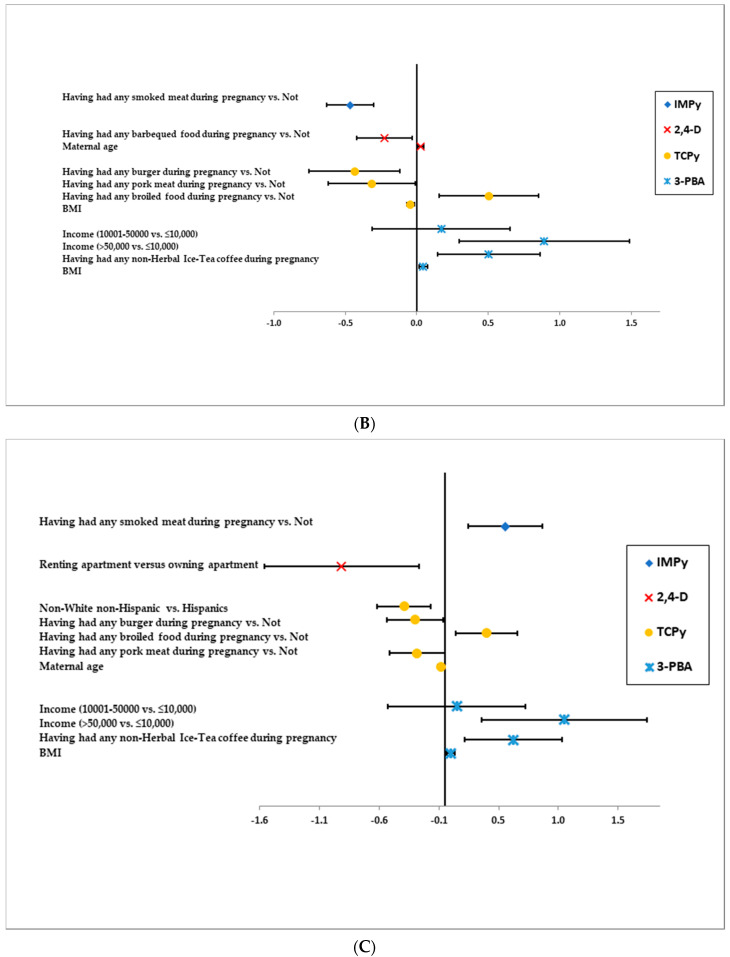
(**A**)**.** Predictors of Concentrations of organophosphate and pyrethroid pesticides in S/H cohort identified with logistic models (≥median vs. <median) (MODEL A) *. (**B**). Predictors of Concentrations of organophosphate and pyrethroid pesticides in S/H cohort identified by linear model with < LOD imputed by LOD/√2 (MODEL B). (**C**). Predictors of concentrations of organophosphate and pyrethroid pesticides in S/H cohort identified by regression models with lower values censored by LOD (MODEL C). BMI: Body mass Index. * No predictors were selected by backward elimination procedure with the model for TCPy.

The predictors of the concentrations of the organophosphate and pyrethroid pesticides are illustrated in Figure 3A–C for the TDID cohort and Figure 4A–C for S/H cohort. In these figures, we present the predictors that were associated with the pesticides at *p* < 0.1 in the final logistic (model A), linear (model B), and regression analysis with data subject to left censoring (model C).

### 3.1. IMPy

In the TDID cohort, consumption of seafoods and butter during pregnancy was associated with higher and lower IMPy concentrations, respectively, obtained from logistic models (model A and model C).

In the S/H cohort, having any smoked meat during pregnancy was associated with higher concentrations of IMPy in models B and C (Figure 4B,C).

### 3.2. 2,4-D

In the TDID cohort, having more than $50,000 annual household income was associated with higher odds of 2,4-D in model A (OR = 4.23; 90%CI:1.89,9.49). Nonetheless, having $10,001–$50,000 annual income was associated with lower concentrations of 2,4-D in model C (b = −0.57; 90%CI: −1.06, −0.08) (Figure 3A,C).

In the S/H cohort, consuming any barbequed food during pregnancy was associated with lower concentrations of 2,4-D in models A and B (Figure 4A,B). Renting households was associated with lower concentrations of 2,4-D only in model C, where <LOD is considered as left-censored (Figure 4C).

### 3.3. TCPy

In the TDID cohort, White and non-White non-Hispanics tended to have higher concentrations of TCPy in all three models compared to Hispanics. Divorced/separated/widowed women tended to have higher concentrations of this chemical compared to never-married women, while married women tended to have lower concentrations of TCPy (model C) (Figure 3C). Having had any processed meat during pregnancy was associated with lower odds of ≥median concentrations of TCPy in model A and lower concentrations of TCPy in model B (Figure 3A,B)

In the S/H cohort, consuming any burgers during pregnancy was associated with lower concentrations of TCPy in models B and C (Figure 3B,C). However, having any broiled food during pregnancy was associated with higher concentrations of TCPy in models B and C (Figure 3A,C). Non-White non-Hispanics in S/H cohort also tended to have lower concentrations of TCPy.

### 3.4. 3-PBA

In the TDID cohort, non-white non-Hispanic women were more likely to have higher concentrations of 3-PBA in models B and C (Figure 3B,C). Having ≥ $50,000 annual household income was associated with higher odds of ≥median concentrations of 3-PBA. Nonetheless, full-time or part-time work during pregnancy was associated with lower odds of ≥ median concentrations of 3-PBA.

Having any organic food during pregnancy was associated with lower concentrations of 3-PBA in model B. Having any low-fat fish during pregnancy was associated with higher odds of ≥median concentrations of 3-PBA from model A.

In the S/H cohort, the women who had ≥$50,000 annual household income were more likely to have higher concentrations of 3-PBA in models B and C. Pre-pregnancy BMI was also associated with slightly higher concentrations of 3-PBA in all three models. Having any non-herbal iced tea during pregnancy was also associated with lower concentrations of 3-PBA (Figure 4B,C). Finally, non-white non-Hispanic women tended to have lower 3-PBA biomarkers compared to Hispanic women.

## 4. Discussion

In this study, we attempted to identify predictors of exposure to diazinon, chlorpyrifos, pyrethroids, and 2,4-D herbicide, and to explore how our results varied depending on the modeling strategies employed. We found that ethnicity, higher income, and high education (at least two years of college) were associated with higher concentrations of most of the pesticides in both cohorts. Higher maternal pre-pregnancy BMI was associated with higher concentrations of 2,4-D and 3-PBA. In both cohorts, demographic predictors of exposure tended to be more robust to different modeling strategies than dietary predictors of exposure.

We used three models to assess the predictors of exposure to the five compounds. Although in most cases, the models yielded similar predictors, there were some clear discrepancies. The discrepancies between modeling strategies were more pronounced in S/H cohort, possibly due to a smaller sample size (*n* = 121). For example, income was associated with 3-PBA in models B and C but not in Model A. The reason for such differences could be loss of information regarding exposure to compounds in the process of dichotomizing the exposure to higher vs. lower level categories in model A. Furthermore, although both Model B and Model C acknowledged that the <LOD observations do not have exact values, model B imputes the <LOD by assigning a constant value to them, while model C incorporates the interval (0, LOD) into the likelihood function for estimating the coefficient of each predictor of pesticides.

The choice of using any of these models depends on the research question. If the predictors of higher concentrations of pesticides are of interest to contrast with lower concentrations, the logistic models would be appropriate. The logistic models could also be used when there are a high number of observations with <LOD concentrations of pesticides. Imputing <LODs using the $\frac{LOD}{\surd 2}$ method allows for the identification of predictors for the continuous measures of each outcome. Nonetheless, the estimated associations could be inaccurate due to their dependency on the imputed values. It is highly unlikely that all the <LOD observations would have equal values in the real world. This method is not recommended when there is a high proportion of <LODs or the distribution is highly skewed that log-normal distribution assumption is questionable [66,68]. Finally, instead of imputing <LOD values, incorporating the interval (0, LOD) into the likelihood for a parametric model allows us to efficiently estimate the model parameters characterizing the effects of predictors. This method depends on the distribution assumption of a parametric model, verifiable by the empirical distribution of the chemical measures estimated using non-parametric likelihood estimation method for data subject to censoring [69]. As we aimed to compare the models across the two cohorts and with other studies we used backward elimination of predictors based on the same preset significance level to generate parsimonious models. In contrast, if the goal of the study is only for prediction, the models will include all the predictors that explain the variability of the concentrations of the chemicals irrespective of the significance level.

We found that the concentrations of the metabolites in both TDID and S/H cohorts were comparable to NHANES 2009–2010 data. Nonetheless, according to a recent study the concentrations of pesticides tends to be lower in US in general compared to developing countries, such as India and Vietnam [70]. These differences in concentrations could be a result of policies to ban a number of pesticides, such as diazinon, or chlorpyrifos in the US [12,13], or different routes of exposure.

We also found differences in the predictors of the five compounds across the cohorts. Although we initially planned to combine the TDID and S/H cohorts by harmonizing the categories, the inherent differences in the population of these cohorts precluded a pooled analysis. The TDID cohort was more racially diverse and resided in all the boroughs in New York City. The participants in S/H cohort, on the other hand, were African-American and Hispanic living in Upper Manhattan and the South Bronx. Our findings suggest significant heterogeneity in the routes of exposure to non-persistent pesticides across populations, although direct comparison is not possible as a different set of potential predictors was collected within each cohort.

In the TDID cohort, White non-Hispanics tended to have higher concentrations of urinary TCPy. Having processed meat or meat during pregnancy tended to reduce the exposures to TCPy, respectively, and each year increase in maternal age was positively associated with exposures to urinary metabolites of 2,4-D.

Interestingly we found that higher income was associated with higher concentrations of 3-PBA, a metabolite of pyrethroids such as permethrin in both S/H and TDID cohort (all models). Our finding was consistent with a similar study conducted in Israel [45]. Higher education and income indicate higher socio-economic standards of living, which could affect diet and other factors predisposing one to higher exposure levels [45,71]. A possible reason for that could be a different frequency of indoor pesticides application in this cohort (Figure 2). There was a higher proportion of participants of the S/H cohort whose income was >$50,000 compared to TDID cohort (Table 1). Higher income is also related to higher socioeconomic class and possibly different dietary habits. Participants from high-income households also tend to have different dietary habits [72,73]. Nonetheless, we did not collect thorough information on the dietary habits of the S/H participants; the questionnaire did not ask about fruit and vegetable consumption.

We found that non-Hispanic women tended to have higher concentrations of 3-PBA and TCPy regardless of their race in TDID cohort. Our findings are consistent with McKelvey, Jacobson, Kass, Barr, Davis, Calafat and Aldous [57] and with those from the Mothers and Newborns cohort in New York City [56], where non-Hispanic African-Americans tended to have higher total concentrations of organophosphates. A common reason for such a finding could be the different frequency of residential application of these pesticides among these populations and the buildings’ condition [56]. Nonetheless, in the S/H cohort we found that Hispanics tended to have higher concentrations of TCPy and 3-PBA. The participants of S/H cohort were selected during their subsequent pregnancy from mothers and newborns cohort in New York. Thus, their distinction from the parent cohort could explain this discrepancy.

We also found consistently higher concentrations of 3-PBA in women with high pre-pregnancy BMI. In Italy, the mean concentration of 3-PBA—a metabolite of permethrin—was slightly higher among older adults with higher BMI [44]. A possible mechanism for this association is the hypothesized obesogenic properties of 3-PBA at lower levels due to lipophilic properties of parent pyrethroid compounds [74].

Drinking non-herbal tea was one of the only significant dietary predictors found to be positively associated with 3-PBA biomarker concentrations in two models (Models A and C). Previous studies suggest that residues of pesticides can persist in samples of teas [75,76,77]. We also found that eating any organic food predicted lower concentrations of IMPy and 3-PBA, metabolites of diazinon, and pyrethroids such as permethrin. These findings were consistent with results from experimental studies of organic diets, where adults and children assigned to receive organic food/diet had lower organophosphate and pyrethroid pesticides level [48,49,50,51]. Mothers who consumed processed meat in TDID cohort and broiled, barbequed food or burgers in S/H cohort tended to have lower concentrations of organophosphate or 2,4-D herbicide. The low stability of organophosphates to food thermal processing could explain this finding [78,79].

The major strength of this study is that we found generally consistent results using three models to understand the predictors of pesticides in a relatively large sample size. However, our study has a number of limitations. First, as the pesticide concentrations were measured to perform secondary analysis in these cohorts, we did not have a robust measure of diet, particularly in the S/H cohort. Fruit and vegetable intake was not measured in either cohort. Second, the exposure was assessed only at one point in time; given the short half-life of these compounds and the fact that spot-urines were collected, it likely does not represent exposure in the entire pregnancy period. A longitudinal study conducted among 19 individuals demonstrated a significant variability in the concentrations of pesticides measured in spot urine [80]. This could introduce misclassification relative to long-term exposure. Third, most of the study participants in both cohorts were of Hispanic ethnic background. Therefore, the findings could not be generalized to the entire population in New York City. Finally, the high number of <LOD observations and small sample sizes limited the robustness of our results.

## 5. Conclusions

In this study, we attempted to estimate the predictors of four pesticides and 2,4-D herbicide using three different modeling strategies. As the purpose of this study was to predict and compare the exposure using three models, we selected the factors that were associated with exposure using the significance level of *p* < 0.1 to keep the predictors in the three models as our criterion. Nonetheless, this criterion should not be encouraged when models are exploring a causal hypothesis or a purely predictive model. The predictors of compounds were generally similar across the models. Nonetheless, the choice of modeling led to a few different predictors. In future studies, the question that the researcher is aiming to investigate should also be considered to inform the choice of models.

## Figures and Tables

**Table 1:** Basic Demographic comparison between Thyroid Disruption and Infant Development (TDID) and Sibling/Hermanos (S/H) cohort.

	S/H Cohort (*n* = 121)	TDID Cohort (*n* = 153)	*p*-Value
	*n*%	*n*%	
**Education**			
Not Completed High School	25 (20.7)	22 (14.4)	0.49
4yr college	32 (26.4)	40 (26.1)	
4 + yr college	29 (24.0)	37 (24.2)	
Completed High School	35 (28.9)	54 (35.3)	
**Marital Status**			
Never Married	41 (33.9)	47 (30.7)	0.09
Married/Lived with same partner > 7yrs	67 (55.4)	99 (64.7)	
Divorced/Widowed/Separated	13 (10.7)	7 (4.6)	
**Household income**			
Less than or = 10,000	21 (16.4)	63 (49.7)	0.0001
10,001–50,000	76 (62.8)	37(24.2)	
>50,000	22 (18.2)	40 (26.1)	
Missing	2	13	
**Smoking Status (pregnancy)**			
Smoker	6 (5.0)	3(2.0)	0.16
Non-smoker	115 (95.0)	150 (98.0)	
**Employment status**			
Employed	68 (56.2)	85 (55.6)	0.91
Unemployed	53 (43.8)	68 (44.4)	
**Race/ethnicity**			
White-nonHispanic	NA	28 (18.3)	NA *
African American-Non-Hispanic	52 (43.0)	10(6.5)	
Asian-nonHispanic	NA	8 (5.2)	
Hispanic	69 (57.0)	107 (69.9)	
	**Mean (SD)**	**Mean (SD)**	
**Maternal pre-pregnancy BMI**	27.2 (6.1)	25.0 (6.4)	0.002
**Maternal Age**	31.2 (4.4)	28.9 (5.5)	0.0006

* As TDID and S/H cohort were inherently different and had distinct inclusion criteria based on neighborhood in New York City, this value is not applicable for ethnicity and race. *p*-values were from Wilcoxon rank sum test and Chi-square test to detect differences between cohorts in continuous and categorical variables, respectively.

**Table 2:** Pesticide Exposure descriptive statistics in TDID and S/H Cohorts.

Biomarker Name	Mean (SD) (µg/g Creatinine) ^a^	Geometric Mean (95%CI) (µg/g Creatinine)	Median (µg/g Creatinine) ^a^	LOD (µg/L)	# of Values > LOD	# of Values < LOD (%)
**TDID cohort**						
IMPY	0.3 (0.4)	NC ^b^	0.2	0.1	62	91 (59.5%)
2,4-D	0.6(1.5)	NC ^b^	0.3	0.15	89	64 (41.8%)
TCPy	1.6 (1.2)	1.1 (1.0,1.3)	1.2	0.1	135	18 (11.8%)
3-PBA	0.9 (3.1)	0.4 (0.3,0.4)	0.3	0.1	113	40 (26.1%)
**S/H cohort**						
IMPY	0.2 (0.4)	0.1 (0.1,0.2)	0.1	0.1	78	43 (35.5%)
2,4-D	0.2 (0.3)	0.2 (0.2,0.2)	0.2	0.15	90	31 (25.6%)
TCPy	1.1 (0.9)	0.8 (0.7,1.0)	0.9	0.1	108	13 (10.7%)
3-PBA	0.7 (1.8)	0.3 (0.2,0.3)	0.3	0.1	89	32 (26.4%)

^a^ Calculated for all the observations and divided by urinary creatinine levels to adjust for urinary dilution. ^b^ Geometric means are not calculated when the detection frequency is <60% (Centers for Disease Control and Prevention, 2019).

## Supplementary Materials

The following are available online at https://www.mdpi.com/1660-4601/17/17/6164/s1, Table S1: Original and Harmonized Response-Levels for TDID and S/H Cohorts, Table S2: Comparison of the demographic characteristics of TDID participants included and not included in the study, Table S3: Pesticide exposure descriptive statistics in NHANES 2001–2002 and 2009–2010 among Females, Figure S1: Comparison of Geometric mean of pesticides in two cohorts.

## Abbreviations

N	Number
USDA	United States Department of Agriculture
NHANES	National Health and Nutrition Examination Survey
SD	Standard Deviation
SE	Standard Error
LOD	Lower than limit of Detection
