# Supplementary material for: Predictors of Urinary Pyrethroid and Organophosphate Compound Concentrations among Healthy Pregnant Women in New York
Source: Int J Environ Res Public Health. 2020 Aug 25;17(17):6164. doi: 10.3390/ijerph17176164 (PMC7504694; doi:10.3390/ijerph17176164)
Supplement: Supplementary file 1 [file ijerph-17-06164-s001.pdf]

Supplementary Table 1. Original and Harmonized Response -Levels For TDID And S/H Cohorts

	S/H Cohort	TDID cohort	Harmonized Variable
	n (%)	n(%)	
**Maternal Education**			
	Less than High School	Less Than Grade 7	Not Completed High School
		Less than grade 8	
	Some High School	Grades 9-12 (partial high school)	
	GED	High school degree (including GED)	Completed High School
	High School diploma		
	Some College	Some college (>1 year) or other specialized or technical training	Attended College
	2yr college		At least 2 years of College
	4yr college	4-year college or university degree	
	4+yr college	Graduate degree (including MD, JD)	
**Marital Status**			
	Married	Married/living with partner	Married/living with partner
	Living with the same partner more than 7 years		
	Divorced	Divorced/widowed/separated	Divorced/widowed/separated
	Widowed		
	Separated		
	Never Married	Never married	Never married
**Household income**			
	Less than 10,000	Less than $10,000	Less than $10,000
	10,001-20,000	$10,000 - $24,999	Up to $50,000
	20,001-30,000		
	30,001-40,000	$25,000 - $49,999	
	40,001-50,000		
	50,001-60,000	$50,000 - $74,999	More than $50,000
	60,001-70,000		
	70,001-80,000	$75,000 - $99,999	
	80,001-90,000	$100,000 - $149,999	

	S/H Cohort	TDID cohort	Harmonized Variable
	> 90,000	>= $150,000	
**Smoking Status (pregnancy)**			
Smoker	Smoke Cigarettes	Smoked Cigarettes	Smoked Cigarettes
Non-smoker	Do not Smoke Cigarettes	Did not Smoke Cigarettes	Did not Smoke Cigarettes
**Employment status**			
		Full-Time	Employed during pregnancy
	Employed during pregnancy	Part-Time	
	Unemployed during pregnancy	Unemployed	Employed during pregnancy
**Race/Ethnic Background**	African American	Black/African American	
	Dominican/Dominican American	White	
		Asian	
		Other	
		Hispanic	
		Non-Hispanic	
	**Dietary predictors**		
**Meat***[a]			
Beef	Never	Never	Did not consumed
Pork	Rarely	<1 times per week	Ever consumed
Poultry	(1/month or less)	1 time per week	
Any other meat	2-3/month	2-3 time per week	
Sausage or bacon	1/week	4-6 time per week	
Fish	2-4 week	1 time a day	
	Daily	> 1 time a day	
	Don't know		

*The questions about meat consumption in S/H cohort also asked about cooking (broiled/cooked/Fried/Barbecued/Cooked with any method appeared brown upon serving). The mode of cooking was not asked in TDID cohort.

[a] Separate variables were created for poultry, beef, pork, processed meat and fish to indicate their consumption.

Supplementary Table 2. Comparison of the demographic characteristics of TDID participants included and not included in the study

Variable	Level	Total N=316 (%, mean +/- SD)	Included N=153	Not Included N=163	p value
Race (n,%)	White	73 (23.1%)	32 (20.92%)	41 (25.15%)	0.79
	Black/African American	20 (6.33%)	11 (7.19%)	9 (5.52%)	
	Native Hawaiian/Pacific Islander	1 (0.32%)	0 (0%)	1 (0.61%)	
	Asian	14 (4.43%)	8 (5.23%)	6 (3.68%)	
	Other	206 (65.19%)	101 (66.01%)	105 (64.42%)	
	Not Reported	2 (0.63%)	1 (0.65%)	1 (0.61%)	
Ethnicity (n,%)	Hispanic/Latino	212 (67.09%)	106 (69.28%)	106 (65.03%)	0.422
	Not Hispanic/Latino	104 (32.91%)	47 (30.72%)	57 (34.97%)	
Marital.Status (n,%)					0.93
	Never married	94 (29.75%)	47 (30.72%)	47 (28.83%)	
	Divorced/widowed/separated	14 (4.43%)	7 (4.58%)	7 (4.29%)	
	Married/living with partner	207 (65.51%)	99 (64.71%)	108 (66.26%)	
	Missing	1 (0.32%)	0 (0%)	1 (0.61%)	
Home Ownership Status (n,%)	Live with parents/other adults	47 (14.87%)	19 (12.42%)	28 (17.18%)	0.08
	Own single family/townhouse/condo	59 (18.67%)	23 (15.03%)	36 (22.09%)	
	Rent	210 (66.46%)	111 (72.55%)	99 (60.74%)	
Household.Income (n,%)	\$10,000 - \$50,000	74 (23.42%)	37 (24.18%)	37 (22.7%)	0.80
	Less than \$10,000	126 (39.87%)	63 (41.18%)	63 (38.65%)	
	More than \$50,000	84 (26.58%)	40 (26.14%)	44 (26.99%)	
	Not reported (or refused to answer)	32 (10.13%)	13 (8.5%)	19 (11.66%)	
Employment.Status (n,%)	Employed	180 (56.96%)	85 (55.56%)	95 (58.28%)	
	Unemployed	136 (43.04%)	68 (44.44%)	68 (41.72%)	
Education (n,%)					
	At least two years of College	112 (35.44%)	54 (35.29%)	58 (35.58%)	0.63
	High school degree (including GED)	75 (23.73%)	40 (26.14%)	35 (21.47%)	
	Less than High School	50 (15.82%)	20 (13.07%)	30 (18.4%)	
	Some college (>1 year) or other specialized or technical training	76 (24.05%)	37 (24.18%)	39 (23.93%)	
	Missing	3 (0.95%)	2 (1.31%)	1 (0.61%)	
Maternal.Age (mean +/- SD)		28.81 (+/- 6.41)	28.86 (+/- 6.38)	28.77 (+/- 6.46)	0.902 (0.796)
BMI (mean +/- SD)		24.69 (+/- 5.07)	25.1 (+/- 5.46)	24.3 (+/- 4.66)	0.17 (0.335)

Footnotes:

[1] For categorical variables Pearson's Chi-Square test was performed if all the cell counts were >5. Otherwise Fisher's exact test was performed.

[2] For numerical variables t-test was used. The p-value in the brackets was calculated using Wilcoxon's Signed Rank test.

Supplementary Table 3-Pesticide exposure descriptive statistics in NHANES 2001-2002 and 2009-2010 among Females

**Metabolite name**	**N**	**Geometric Mean(SE)**	**Median**	**N**	**Geometric Mean(SE)**	**Median**
	**2001-2002**			**2009-2010**		
IMPy	1617	NC*	<LOD	1404	<LOD	<LOD
2,4-D	1539	NC	<LOD	1343	.347 (.298-.404)	.320 (.270-.370)
TCPy	1595	1.40 (1.21-1.62)	1.63 (1.32-2.01)	1404	.704 (.631-.786)	.940 (.830-1.04)
3-PBA	1619	.311 (.260-.371)	.250 (.200-.320)	1392	.415 (.366-.471)	.400 (.350-.480)

*NC not calculated due to high number of participants with <LOD.(Centers for Disease Control & Prevention, 2009)

Supplementary Figure 1. Comparison of Geometric mean of pesticides in two cohorts

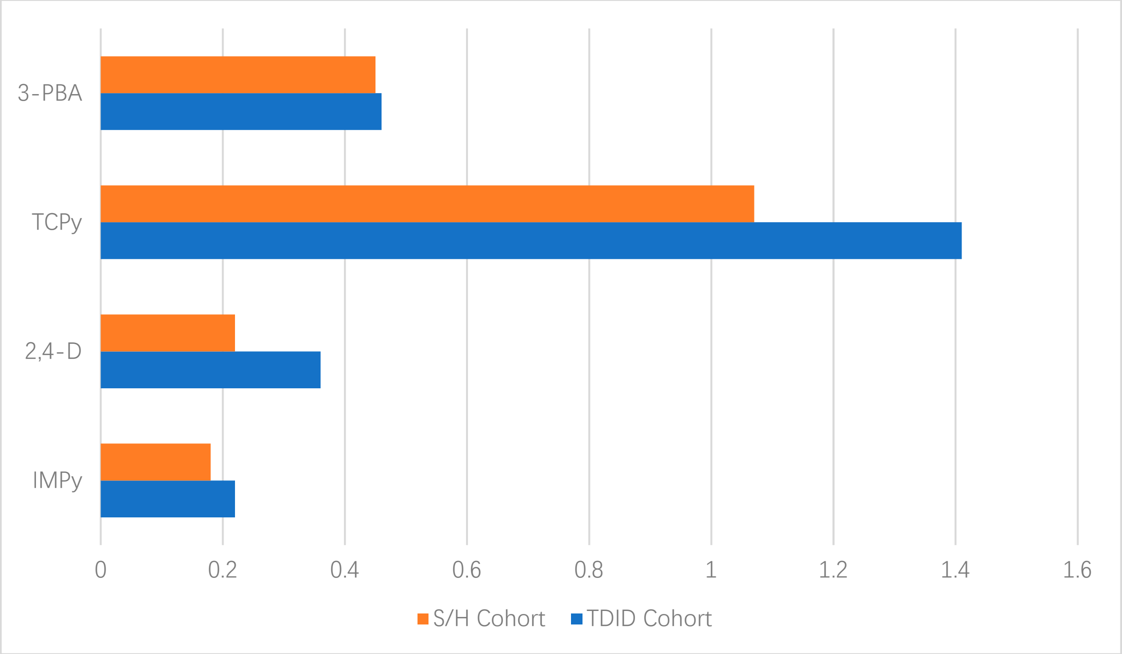

Centers for Disease Control & Prevention. (2009). Fourth national report on human exposure to environmental chemicals. In: Atlanta, GA.
